# AP-3 adaptor complex-mediated vesicle trafficking

**DOI:** 10.52601/bpr.2021.200051

**Published:** 2021-04-30

**Authors:** Zhuo Ma, Md. Nur Islam, Tao Xu, Eli Song

**Affiliations:** 1 National Laboratory of Biomacromolecules, CAS Center for Excellence in Biomacromolecules, Institute of Biophysics, Chinese Academy of Sciences, Beijing 100101, China; 2 University of Chinese Academy of Sciences, Beijing 100049, China; 3 Guangzhou Regenerative Medicine and Health Guangdong Laboratory (Bioland Laboratory), Guangzhou 510005, China

**Keywords:** AP-3, Vesicle trafficking, Cargo sorting, Disease/disorder

## Abstract

The transport of cargo proteins to specific subcellular destinations is crucial for the different secretory and endocytic traffic pathways. One of the most important steps in maintaining the accuracy of this process is the recruitment of adaptor protein (AP) complexes to the membrane for recognizing and packaging cargo proteins into nascent vesicles. Adaptor protein complex 3 (AP-3) is a heterotetrametric complex implicated in the trafficking of cargo proteins from the trans-Golgi network (TGN) and/or endosomes to lysosomes or lysosome-related organelles (LROs). This complex is also involved in the biogenesis of synaptic vesicles (SVs) in neurons and of dense core vesicles (DCVs) in endocrine cells as well as in the recycling of receptors in immune cells and the regulation of planar cell polarity (PCP) proteins. Functional defects in AP-3 cause multiple abnormalities in cellular vesicle trafficking and related organelle function, leading to various disorders, such as Hermansky-Pudlak syndrome (HPS). However, the molecular mechanism underlying AP-3 has not been fully elucidated, and further investigations are needed to understand AP-3-mediated trafficking, its associated molecules and its related roles in inherited diseases. Here, we review the current understanding of AP-3 in cellular vesicle trafficking, especially focusing on mammalian systems.

## INTRODUCTION

The trafficking of proteins and other macromolecules is one of the most essential life activities performed by cells. Eukaryotic cells have various organelles surrounded by specific membranes that perform different functions. In the endocytic and exocytotic pathways, the trafficking of cargoes between organelles or between organelle and cell membrane is mainly mediated by membrane-encapsulated transport carriers, generally known as vesicles. Briefly, vesicles are formed when cargoes are recruited to the donor membrane and packed into a nascent vesicle, which is then pinched off from the donor membrane. The free vesicles are then transported through the cytosol to the destination membrane and fuse with the target membrane compartment, achieving the transport and exchange of materials (Park and Guo 2014). The trans-Golgi network (TGN) is generally considered a central station for cargo sorting where proteins and other molecules are sorted into distinct vesicles and further delivered to their appropriate intracellular destinations (Guo *et al*. 2014). One key group of players that function in the sorting process are adaptors, which localize at the donor membranes and recruit cargoes by direct or indirect interactions. Adaptor protein (AP) complexes are a well-known family of adaptors that play a crucial role in cargo selection and vesicle formation. AP complexes specifically recognize and interact with the sorting signals in the cytoplasmic domains of transmembrane cargo proteins or cargo receptors, and they recruit clathrin or other accessory proteins and then package selected cargoes into a departing vesicle (Park and Guo 2014).

**Figure 1:**
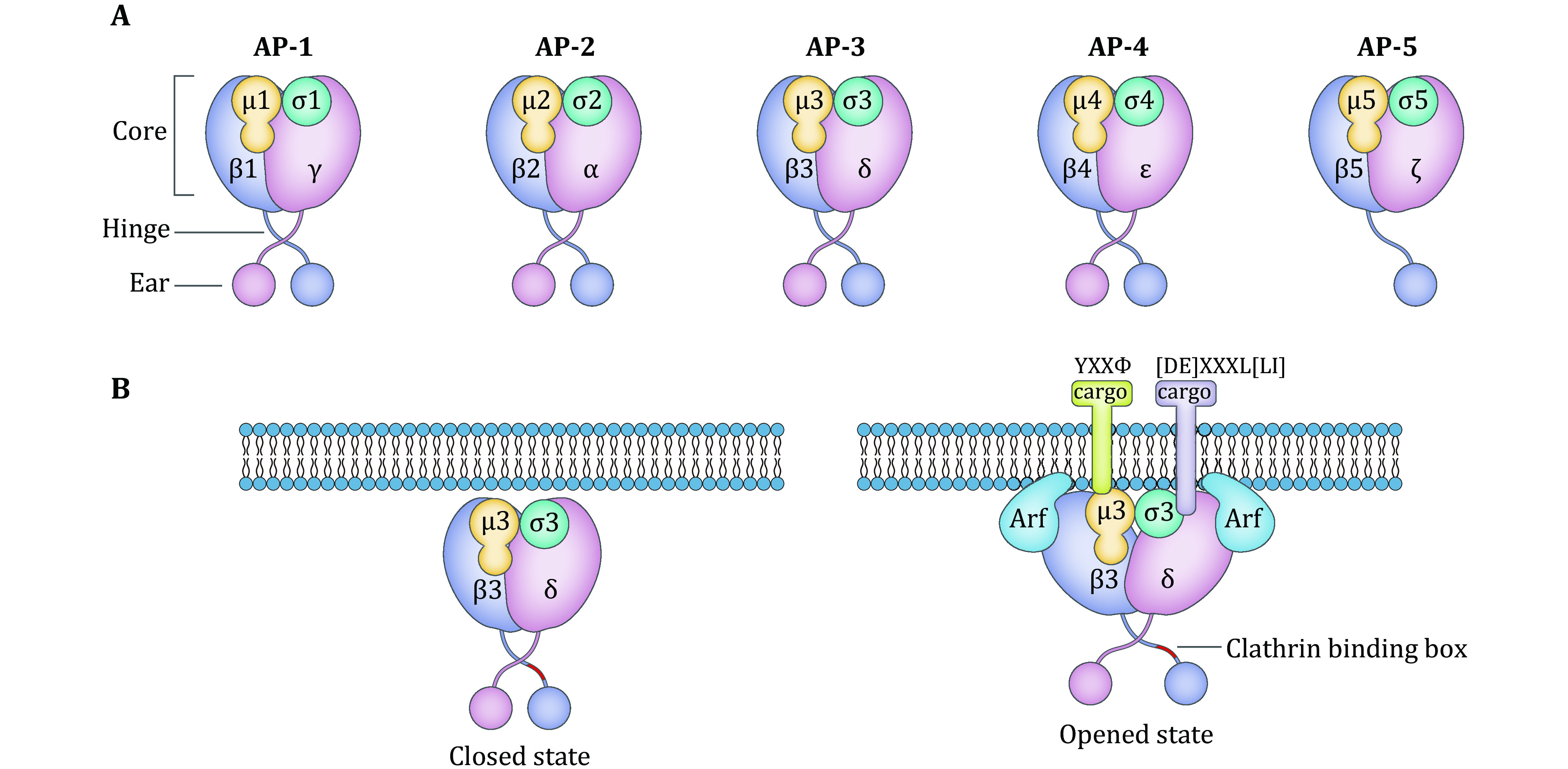
Schematic representation of AP complexes. **A** Diagrams of heterotetrameric AP complexes. Subunit names are indicated, and homologous subunits are depicted in the same color. All AP complexes consist of core, hinge and ear domains, except AP-5, which lacks the hinge domain. **B** The schemes represent the closed and opened state of the AP-3 complex. Based on the known structure of the AP-1 core domain, the cargo binding sites of AP-3 are hindered in the locked state. In the presence of ARFs, AP-3 is recruited to the membrane and undergoes a conformational change to the opened state when both the YXXΦ and [DE]XXXL[LI] motif-binding sites are exposed and recognize the cargo proteins with consensus motifs

To date, five members of the mammalian AP complex family have been identified, namely, AP-1, AP-2, AP-3, AP-4 and AP-5. These molecules are heterotetrametric complexes, each of which contains four subunits, including one small (σ1–σ5), one medium (μ1–μ5) and two large (γ/α/δ/ε/ζ and β1–β5) subunits, which form a central brick-like core and two smaller globular appendages connected by flexible linkers (Fig. 1A). One of the large subunits (γ/α/δ/ε) is responsible for interacting with the target membrane, and the other large subunit (β1–β3) recruits the clathrin coat protein via a clathrin-binding motif (Boehm and Bonifacino 2001; Ohno 2006; Park and Guo 2014). An exception is the β4 subunit of AP-4, which lacks the clathrin-binding box and is thus not capable of binding to clathrin (Dell'Angelica *et al*. 1999a). The N-terminal domains of the two large subunits, the small subunit and the medium subunit form the core brick-like domain, which interacts with cargo molecules, including adenosine diphosphate (ADP)-ribosylation factor (ARF) proteins and phospholipids. In contrast, the C-termini of the two large subunits are appendage domains that interact with a series of accessory proteins. The medium subunit (μ) is responsible for cargo recognition, and the small subunit (σ) is proposed to stabilize the complex (Park and Guo 2014).

The five AP complexes have different subcellular localizations and distinct functions. The most studied and well-known AP complex variant is AP-2, which is related to clathrin-mediated endocytosis from the plasma membrane, while the other adaptor proteins mainly participate in cargo sorting from the TGN or endosomes to lysosomes or other organelles (Collins *et al*. 2002; Ohno 2006; Park and Guo 2014). Here, we review the recent advances regarding the structure, recognition of sorting signals, coat proteins, associated lipids, subcellular localization, cellular trafficking, biological function and associated diseases of AP-3 mainly in mammals.

## STRUCTURE OF AP-3

Similar to all AP complexes, AP-3 is composed of four subunits as follows: two large subunits (δ and β3), one medium subunit (μ3) and one small subunit (σ3). These subunits assemble into a central core and two appendage domains linked by flexible linkers, also known as the hinge region (Ohno 2006; Park and Guo 2014) (Fig. 1B)**.** The large subunit contains three domains as follows: the N-terminal domain, which interacts with the μ and σ subunits to form the core; the C-terminal appendage, also called the ear domain; and the hinge region, which connects the above two domains. The medium μ subunit includes two domains as follows: one is the N-terminal domain, which constitutes one-third of the polypeptide and contributes to the formation of a part of the core; and the other is the C-terminal domain, which constitutes the remaining two-thirds of the polypeptide and mediates cargo recognition by directly binding to the sorting signals of cargo proteins (Mardones *et al*. 2013). The small σ subunit is also a part of the core and is implicated in the stabilization of the complex as suggested by its X-ray crystal structure (Collins *et al*. 2002). The N-terminal domains of the μ subunit and σ subunit have some similarities, and together they stabilize the complex (Ohno 2006).

Multiple isoforms of the AP-3 subunits encoded by different genes have been identified as follows: the β subunit of AP-3 has two isoforms (β3A and β3B); the μ subunit has two isoforms (μ3A and μ3B); and the σ subunit has two isoforms (σ3A and σ3B). The σ3A and σ3B isoforms are ubiquitous and are functionally equivalent, and the β3B isoform is mostly expressed in neuronal tissues (Danglot and Galli 2007). In theory, eight types of AP-3 heterotetramers can be assembled by different combinations of the various subunits (Mattera *et al*. 2011). In practice, two different isoforms of the AP-3 complex have been identified *in vivo* as follows: AP-3A (consisting of δ, β3A, μ3A and σ3 (A or B)) is ubiquitous; and AP-3B (consisting of δ, β3B, μ3B and σ3 (A or B)) is neuron specific (Nakatsu *et al*. 2004; Park and Guo 2014).

## RECOGNITION OF SORTING SIGNALS BY AP-3

As adaptors, AP complexes specifically select and interact with cargoes or cargo receptors via recognition of the signals within the sequences of cargo proteins. These signals are generally short, linear consensus sequences of amino acid residues located in the cytoplasmic region of transmembrane proteins or cargo receptors (Park and Guo 2014). To date, there are two well-characterized recognition motifs as follows: the YXXΦ motif (where X represents any amino acid and Φ represents an amino acid containing a bulky hydrophobic residue), a tyrosine-based sorting motif that directs endosomal and lysosomal targeting and sorting at the TGN; and the [DE]XXXL[LI] consensus motif, a dileucine-based motif that is mainly present in the TGN or endosomal system (Bonifacino and Dell'Angelica 1999; Ohno 2006; Guo *et al*. 2014). These motifs are not exactly conserved in every site but are instead degenerate motifs containing four to seven residues, in which two or three key sites are critical for producing the functional characteristics of these motifs (Bonifacino and Lippincott-Schwartz 2003). The critical residues are usually bulky and hydrophobic, while charged residues are also important determinants of the specificity for certain signals and contain functional significance for the trafficking of membrane proteins (Kirchhausen 1999; Bonifacino and Traub 2003).

The AP-3 complex recognizes both the YXXΦ and [DE]XXXL[LI] motifs via the µ3 subunit and the combination of the δ-σ3 subunits, respectively (Park and Guo 2014). The YXXΦ binding site on μ3 is similar to the binding sites on the AP-1 μ1 subunit and the AP-2 μ2 subunit (Owen and Evans 1998; Mardones *et al*. 2013). The mechanism by which AP-3 recognizes the [DE]XXXL[LI] motif and the corresponding conformational change it must undergo remain to be characterized. Additionally, other noncanonical recognition consensus and mechanisms need to be identified.

## COAT PROTEINS OF AP-3

Coat protein complexes, such as COPI, COPII and clathrin, are involved in the transportation of cargo proteins to different cellular compartments. Unlike the well-known association of clathrin with the AP-1 and AP-2 complexes, the concept of coat proteins associated with AP-3 remains controversial. The δ subunit of AP-3 is responsible for binding to the target membrane, and the β3 subunit recruits clathrin via the clathrin-binding box, LΦXΦD/E, which resides in the hinge region of the complex (Owen *et al*. 2004). The interaction between AP-3 and clathrin has been verified by a coassembly assay, and cellular AP-3 has been demonstrated to colocalize with clathrin via immunoelectron and immunofluorescence microscopy (Dell'Angelica *et al*. 1998). Furthermore, an *in vitro* binding experiment has demonstrated that the appendage domain of the β3 subunit of mammalian AP-3 associates with the N-terminal domain of the clathrin heavy chain (Dell'Angelica *et al*. 1998). These findings suggest that AP-3 has the potential to play a role in protein-sorting events as a clathrin-associated adaptor in mammals. Colocalization analysis of AP-3 and AP-1 with clathrin by dual color labeling analysis has demonstrated that AP-3 colocalizes with clathrin on the vesicular profile when the vesicle buds from the donor membrane but to a lesser degree than AP-1 (Peden *et al*. 2004). However, the lack of AP-3 in purified clathrin-coated vesicles implies that AP-3 might work independently of clathrin, at least in some cases (Dell'Angelica *et al*. 1997b). Zlatic *et al*. demonstrated that the association of AP-3 with clathrin is dispensable for endosomal AP-3 vesicle biogenesis by rapid chemical-genetic perturbation of clathrin function in PC12 cells, indicating that endosomal AP-3-clathrin interactions fulfill functions distinct from AP-1 and AP-2 (Zlatic *et al*. 2013). In addition to clathrin, VPS41, a member of the homotypic fusion and vacuole protein sorting (HOPS) complex, which includes a single clathrin heavy chain repeat in its C-terminal domain, also reversibly self-assembles into a lattice with AP-3 and promotes DCV biogenesis by a conserved mechanism. This finding suggests that VPS41 has the potential to act as a coat protein of AP-3 and take part in cargo sorting in the regulated secretory pathway (Asensio *et al*. 2013).

## ASSOCIATED LIPIDS OF AP-3

Lipid function and switching are believed to play crucial roles in cargo sorting and transport during vesicular trafficking. In the secretory pathway, lipids are involved in the entire journey of the secretory vesicles, including the formation of budding sites at the donor membrane, protein recruitment at the budding sites and formation of exocytotic sites as well as docking, priming and fusion with the target membrane (Tanguy *et al*. 2016). Although a direct interaction between AP-3 and lipids has not yet been identified, a relationship between AP-3 and its related lipids could be proposed based on the distribution and roles of different lipids.

Phosphatidylinositol 3-phosphate (PI3P) is detected predominantly on early endosomes, while cytoplasmic lipids, such as PI(3,5)P_2_ and bis-monoacylglycerophosphate (BMP), are enriched in late endosomes and lysosomes. The plasma membrane is enriched with phosphoinositides, such as PI4P, PI(3,4)P_2_, PI(4,5)P_2_, PI(3,4,5)P_3_, phosphatidylserine (PS) and phosphatidylethanolamine (PE) (De Craene *et al*. 2017). These phosphoinositides specifically interact with coat proteins or accessory proteins and recruit organelle-specific effector proteins that are involved in vesicle trafficking and signal transduction on the cytosolic surface (Van Meer and de Kroon 2011; Tanguy *et al*. 2016). The Golgi membrane of mammalian cells contains the same lipids as the plasma membrane, but the proportions are quite different. Two leaflets of the Golgi membrane bilayers, the luminal and cytosolic leaflets, contain different types of lipids. The cytosolic leaflet of the Golgi apparatus contains DAG, PA and phosphoinositides, which are involved in the formation of secretory vesicles (Van Meer and de Kroon 2011; Tanguy *et al*. 2016). The TGN membrane is enriched with PI4P, which recruits AP-1 to the Golgi apparatus through direct interaction as identified by *in vitro* assays (Wang *et al*. 2003). Structural analysis has further confirmed that AP-1 interacts with PI(4)P via the binding site localized at its γ subunit (Heldwein *et al*. 2004). Because AP-3 localizes at the TGN, it has a high probability of interacting with PI4P, similar to AP-1; this potential interaction may be responsible for the different cargo sorting and transportation pathways from the TGN. The role of the inositol lipid and its ligand, PI(3,4,5)P_3_, in the regulation of AP-3 function further implies that lipids play important roles in AP-3 function and AP-3–mediated vesicle trafficking (Hao *et al*. 1997).

## SUBCELLULAR LOCALIZATION AND MEMBRANE RECRUITMENT OF AP-3

In mammalian cells, AP-3 was identified as puncta localized to the Golgi region and other peripheral structures as determined by immunofluorescence experiments (Dell'Angelica *et al*. 1997a; Simpson *et al*. 1997). EM localization data also demonstrate a dual localization of the AP-3 complex in the TGN and in endosomes (Simpson *et al*. 1996; Dell'Angelica *et al*. 1998). Peripheral structures partially colocalize with the endosomal marker, transferrin receptor (TfR), indicating that AP-3 exists in some endosomal locations (Dell'Angelica *et al*. 1997a). Within the endosome portion, AP-3 is preferentially presented on tubular endosomal compartments and is particularly associated with distinct buds of endosomal tubules (Peden *et al*. 2004). For the different isoforms of AP-3, the ubiquitously expressed AP-3A localizes to the TGN and the peripheral region where endocytic materials are contained (Dell'Angelica *et al*. 1997b). Neuron-specific AP-3B is associated with endosomes and is involved in the biogenesis of SVs from endosomes as identified by studies on PC12 cells (Faundez and Kelly 2000; Blumstein *et al*. 2001; Nakatsu *et al*. 2004; Zlatic *et al*. 2013). In addition, AP-3 is localized in the Golgi apparatus in neurons and is responsible for the transport of cargoes from the Golgi apparatus to the axon (Li *et al*. 2016).

The mechanism underlying the recruitment of AP complexes and coat proteins to specific membranes has been partially identified and described. The recruitment process involves several common basic mechanisms. Among the five AP complexes, AP-1, AP-3 and AP-4 have similar mechanisms that use the small GTPase, ARF1, which facilitates the activation and recruitment of adaptor proteins, coat proteins and other effectors (Donaldson and Jackson 2011). Initially, the binding of AP-1 to ARF1 induces a change in AP-1 from a closed to an open conformation followed by exposure of the binding sites and a change to a coplanar configuration to allow simultaneous interactions with cargo and clathrin. This interaction further stabilizes the open conformation and brings the complex into close contact with the membrane (Ren *et al*. 2013). AP-3 has a similar localization to AP-1 on the TGN membrane; thus, the mechanism underlying AP-3-dependent protein sorting also relies on the action of kinases, coat or accessory proteins and small GTPases (Fig. 1B). ARF1 regulates the recruitment of AP-3 to membranes, which requires the conversion of ARF1 from a GDP-bound form to a GTP-bound form catalyzed by guanine nucleotide exchange factor (GEF). GTP-bound ARF1 binds to AP-3 and other effectors at the correct membrane with high affinity (Ooi *et al*. 1998). Biochemical evidence and *in vivo* studies also demonstrate that ARF1 promotes the membrane recruitment of AP-3 (Ooi *et al*. 1998). As indicated by an *in vitro* binding assay, ARF6 interacts with the β3 and δ subunits of AP-3, but whether ARF6 is involved in the recruitment of AP-3 to the membrane is still controversial (Ooi *et al*. 1998; Austin *et al*. 2002).

## AP-3 MEDIATED CELLULAR TRAFFICKING

**Figure 2:**
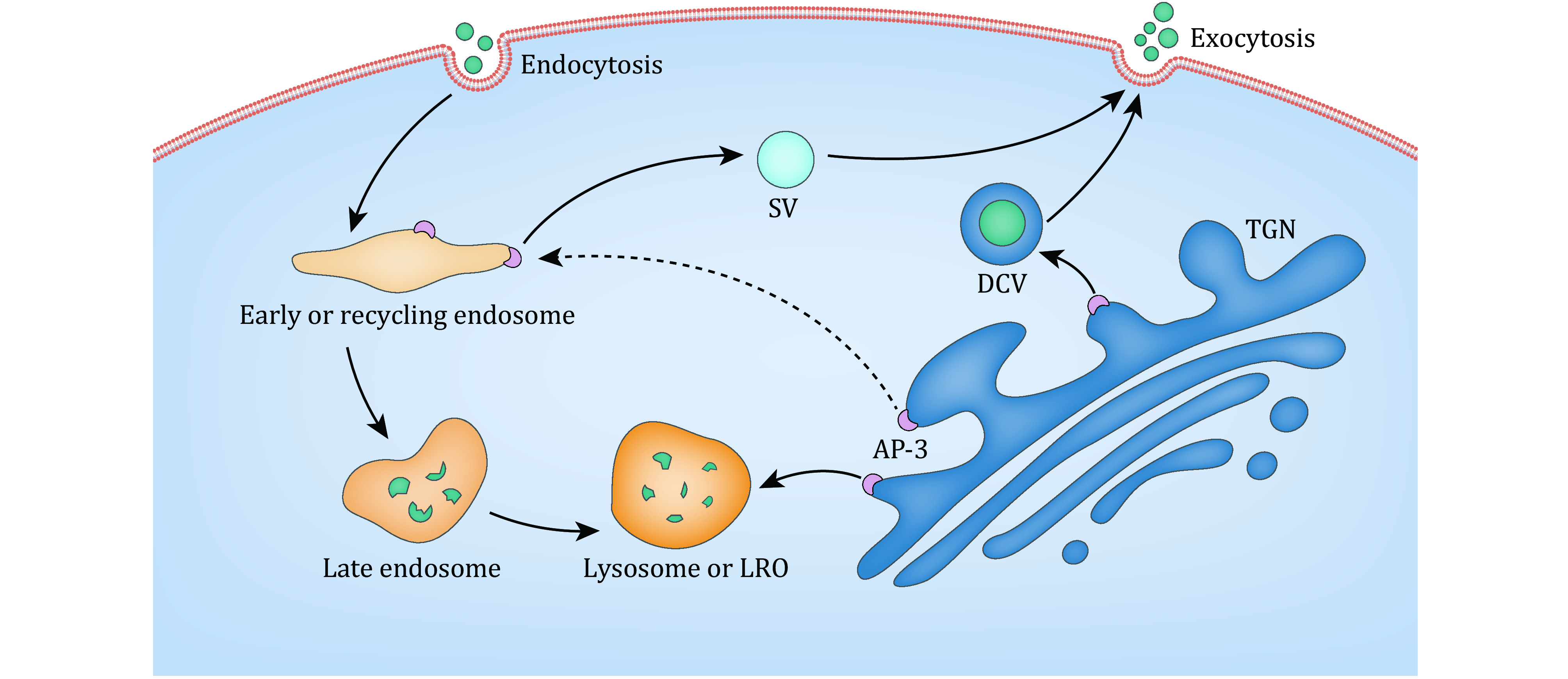
Schematic representation of AP-3 subcellular localization and mediated trafficking. The AP-3 complex localizes to the TGN, early endosomes and recycling endosomes, and it is responsible for regulation of the trafficking of cargo proteins from the TGN and/or endosomes to lysosomes or LROs. The AP-3 complex is also responsible for the biogenesis of SVs in neurons and of DCVs in endocrine cells, and it is involved in the recycling of receptors in immune cells and the regulation of PCP proteins. The corresponding AP-3-mediated trafficking pathways are indicated by arrow lines

By recruiting cargoes and interacting with coated or accessory proteins and lipids, AP-3 participates in the transport of both membrane and molecules from the TGN and/or endosomes to lysosomes or lysosome-related organelles (LROs) (Fig. 2). Inhibition of AP-3 function by RNAi leads to a misrouting of both LAMP1 and LAMP2 to the cell surface (Groux-Degroote *et al*. 2008). This result has been further confirmed in AP-3-deficient cells, in which LAMP1, LAMP2 and melanosomal membrane protein tyrosinase are mislocalized (Dell'Angelica *et al*. 1999b). OCA2, the product of the gene oculocutaneous albinism type 2, resides in the endoplasmic reticulum, and it also localizes to melanosomes. OCA2 contains the dileucine-based signal, LL1, and is an AP-3 cargo according to *in vitro* binding experiments (Sitaram *et al*. 2009). Tyrosinase-related protein 1 (Tyrp1), also known as TRP1, is significantly decreased in AP-3-deficient primary melanocytes. This phenomenon is suppressed by treatment with a lysosome protease inhibitor, indicating that TRP1 is missorted to lysosomes for degradation when AP-3 is absent (Di Pietro *et al*. 2006). However, TRP1 does not directly interact with AP-3 but instead acts through an association with tyrosinase (Kobayashi and Hearing 2007). AP-3 physically interacts with biogenesis of lysosome-related organelles complex-1 (BLOC-1), and simultaneous deficiencies in both AP-3 and BLOC-1 affect the targeting of LAMP1, PI4KIIα and VAMP7 (Salazar *et al*. 2006). The δ subunit of AP-3 interacts with the v-SNARE protein, VAMP7, and mislocalization of the δ subunit causes diminished lysosomal secretion as well as several neurological problems (Martinez-Arca *et al*. 2003). Deletion of AP-3 in yeast is associated with mislocalization of the Vamp3p t-SNARE protein, vacuolar proteins and alkaline phosphatase (Cowles *et al*. 1997).

In addition, AP-3 is involved in the biogenesis of SVs from endosomes in neurons. *In vitro* experiments show that only the neuronal AP-3B generates synaptic vesicles from endosomes although it is not the predominant form of AP-3 in brain (Blumstein *et al*. 2001). In *mocha* mice, an AP-3-deficient mouse model, zinc transporter 3 (ZnT3) and chloride channel (ClC-3) are reduced in SVs, suggesting that the trafficking of ZnT3 and CIC-3 to SVs is regulated by AP-3 (Salazar *et al*. 2004a, b). Hippocampal neurons lacking neuronal AP-3 have diminished asynchronous release, which is accomplished by SVs and impairs the precision of action potential timing (Evstratova *et al*. 2014). In midbrain dopamine neurons, loss of AP-3 reduces vesicular monoamine transporter 2 (VMAT2) localization to release sites and impairs the release of dopamine vesicles (Silm *et al*. 2019). AP-3 targets vesicular glutamate transporter (VGLUT) to SVs with low release probability, suggesting its involvement in VGLUT recycling (Evstratova *et al*. 2014; Li *et al*. 2017). Calcyon, a single transmembrane protein that binds to clathrin light chain in neurons, directly interacts with AP-3 μ3 subunit, and it regulates the trafficking of SVs and the targeting of AP-3 cargoes, including ZnT3 and PI4KIIα (Muthusamy *et al*. 2012).

AP-3 is also involved in the exocytosis of DCVs in endocrine cells. The neuroendocrine cells and endocrine cells from *mocha* mice have increased constitutive exocytosis of DCV cargoes, including soluble cargoes and membrane proteins, but they have a decreased response to stimulation, suggesting that AP-3 deficiency potentially leads to a disturbance of DCV biogenesis in the regulated exocytosis (Grabner *et al*. 2006). In PC12 cells, loss of AP-3 impairs DCV formation and substantially decreases synaptotagmin 1, indicating that AP-3 may concentrate the cargoes required for regulated exocytosis (Asensio *et al*. 2010).

AP-3 is also involved in the recycling of receptors in immune cells and the regulation of planar cell polarity (PCP) proteins. TLR2 stimulation and cytokine activation are involved in the inflammatory responses in innate immunity. AP-3 is required for the trafficking of TLR2 and its ligands to lysosomes for degradation (Petnicki-Ocwieja *et al*. 2015). The trafficking of human CD1b is related to the lipid antigen-presenting process from the sorting of endosomes to late endosomes; this process is mediated by AP-3 (Briken *et al*. 2002). PCP is established by the polarized and asymmetric localization of several membrane PCP proteins, including Vangl2 and Frizzled6. The subcellular localization of Vangl2 is abnormal in AP-3-depleted cells and *mocha* mice, causing auditory and vestibular dysfunction in mutant mice (Tower-Gilchrist *et al*. 2019).

Proteomics and proximity strategies have been used to identify the potential cargoes involved in AP-3-dependent trafficking. Purification of AP-3-dependent vesicles from PC12 cells using ZnT3, a known AP-3 cargo, identified enriched components, including AP-3 subunits, BLOC-1 and PI4KIIα (Salazar *et al*. 2004b). Combination of organelle density gradients and proteome analysis characterized the AP-3-dependent cargo protein profile in the Arabidopsis AP-3β mutant, indicating disturbed aquaporins and phosphorylation patterns (Pertl-Obermeyer *et al*. 2016). However, more research is needed to unravel the precise mechanism underlying the interaction between AP-3 and the newly identified cargoes.

## PHYSIOLOGICAL FUNCTIONS AND ASSOCIATED DISEASES OF AP-3

Functional defects in AP complexes will cause incorrect localization of cargo proteins, subsequently affecting a wide range of cellular activities, including cell signal transduction, organelle dynamics and cellular homeostasis (Park and Guo 2014). The physiological roles of AP-3 have been identified through genetic studies and related inherited diseases. We summarized the related diseases in fruit fly, mouse, dog and human caused by the mutations in different subunits of AP-3 (Table 1).

**Table 1:** Diseases and genetic disorders caused by AP-3 mutations

AP-3 subunit	Species	Disease or disorder	Relevant organelle	References
δ	Fruit fly	Defects in eye pigmentation granule biogenesis	Pigment granules	Ooi *et al*. 1997; Kantheti *et al*. 1998
	Mouse	Diluted coat color, abnormalities in lysosomal enzyme secretion, epileptic seizures, vestibular dysfunction	Melanosomes; Platelet dense granules	Kantheti *et al*. 1998; Ammann *et al*. 2016
	Human	Hermansky-Pudlak syndrome-10 (HPS10), characterized by albinism, early-onset seizures, neurodevelopment delay, infection susceptibility and neutropenia	Melanosomes; Platelet dense granules	Ooi *et al*. 1997; Ammann *et al*. 2016
β3A	Mouse	Color defects, missorting of lysosomal proteins, abnormal LROs, melanosomes and pigment defects	Melanosomes; Platelet dense granules	Feng *et al*. 1999
	Dog	Decreased coat pigmentation, cyclic hematopoiesis	Melanosomes; Platelet dense granules	Dell'Angelica *et al*. 1999b; Benson *et al*. 2003
	Human	Hermansky-Pudlak syndrome-2 (HPS2), characterized by oculocutaneous albinism and platelet storage pool deficiency, immunodeficiency, and pulmonary fibrosis	Melanosomes; Platelet dense granules; Lytic granules	Dell'Angelica *et al*. 1999b; Benson *et al*. 2003; Clark *et al*. 2003
β3B	Mouse	Seizures as well as behavioral problems.	Synaptic vesicles	Seong *et al*. 2005
	Human	Early-onset epileptic encephalopathy (EOEE), characterized by seizures, interictal epileptiform activity, developmental regression or intellectual disability, and onset before 1 year of age	Synaptic vesicles	Assoum *et al*. 2016
µ3B	Mouse	Spontaneous seizure without any deafness or balance problems	Synaptic vesicles	Nakatsu *et al*. 2004

The large δ subunit of AP-3 is defective in the fruit fly *garnet* eye color mutant, which results in a reduced level of pigment granules, indicating that AP-3 is involved in the biogenesis and sorting pathway of pigment granules (Ooi *et al*. 1997). *Mocha* mice, which have δ subunit mutations, present with color defects, inner ear degeneration and neurologic defects, which are associated with abnormalities in cargo sorting from the TGN to the LROs and in SV transport (Kantheti *et al*. 1998). In humans, mutation of the δ subunit causes destabilization of AP-3 in cells and leads to Hermansky-Pudlak syndrome-10 (HPS10), a novel type of HPS with severe neurologic involvement. Using retroviral reconstitution in the patients’ T cells may restore the formation of AP-3 and reverse degranulation defects (Ammann *et al*. 2016).

Mutation of the β3A subunit in dogs causes various phenotypes characterized by canine cyclic hematopoiesis (gray collie syndrome) and decreased coat pigmentation (Benson *et al*. 2003). Genetic deficiencies of the β3A subunit in *pearl* mice present as color defects, abnormal LROs, melanosomes and platelet granules without any neurological defects (Feng *et al*. 1999). In humans, β3A mutation causes Hermansky-Pudlak syndrome-2 (HPS2), a rare autosomal recessive disorder characterized by oculocutaneous albinism and prolonged bleeding (Dell'Angelica *et al*. 1999b). In these cases, the biogenesis and function of LROs, including melanosomes and platelet granules, are abnormal in AP-3-deficient cells. Additionally, AP-3 deficiency leads to increased expression of LAMP-1, LAMP-2 and CD63 on the cell surface of fibroblasts; these markers normally localize to late endosomes or lysosomes (Dell'Angelica *et al*. 1999b). Although CD63 mislocalizes to the cell membrane, perforin and granzymes are correctly localized to the lytic granules in cytotoxic T lymphocytes (CTLs). However, these lytic granules in AP-3-deficient CTLs are unable to move along microtubes towards the docking domain of immunological synapses, thereby preventing their secretion, resulting in no CTL-mediated killing (Clark *et al*. 2003).

Knockout of neuronal β3B in mice results in several neurological and behavioral impairments (Seong *et al*. 2005). The β3B mutation in humans causes severe developmental delay, poor visual contact with optic atrophy and postnatal microcephaly, called early-onset epileptic encephalopathy (EOEE) (Assoum *et al*. 2016).

In addition, µ3B subunit-deficient mice show spontaneous seizures without any deafness or balance problems (Nakatsu *et al*. 2004). To the best of our knowledge, the diseases caused by mutations in other subunits have not yet been reported and remain to be further determined.

## CONCLUSION AND PERSPECTIVES

Over the last several decades, research on AP-3 has expanded to a great extent, and many experimental approaches have been employed to understand the function and molecular mechanism of AP-3 in cellular trafficking. The biological significance of AP-3 has been well identified, but the following aspects remain unclear: the trafficking steps involved from the TGN to lysosomes or other organelles; the fate of AP-3 during vesicle maturation; the associated coat proteins other than clathrin; the associated lipids and their functions in AP-3 trafficking; and the recycling pathway of AP-3. The use of combinations of powerful biochemical methods, genetic tools and imaging techniques, including electron microscopy and super-resolution microscopy, holds great promise for resolving these unknowns that remain in this field. Thus, a more thorough understanding of AP-3 will shed light on the cellular functions and biological significance of the AP-3 complex.

## Abbreviations

**Table 2:** 

ADP	Adenosine diphosphate
AP	Adaptor protein
ARF	ADP-ribosylation factor
BLOC-1	Biogenesis of lysosome-related organelles complex-1
BMP	Bis-monoacylglycerophosphate
ClC-3	Chloride channel 3
DAG	Diacylglycerol
DCV	Dense core vesicles
EOEE	Early-onset epileptic encephalopathy
GEF	Guanine nucleotide exchange factor
GTP	Guanosine triphosphate
HOPS	Homotypic fusion and vacuole protein sorting complex
HPS	Hermansky-Pudlak syndrome
LAMP1	Lysosome-associated membrane glycoprotein 1
LAMP2	Lysosome-associated membrane glycoprotein 2
LRO	Lysosome-related organelle
OCA2	Oculocutaneous albinism type 2
PCP	Planar cell polarity
PE	Phosphatidylethanolamine
PI(3,4)P_2_	Phosphatidylinositol 3,4-bisphosphate
PI(3,4,5)P_3_	Phosphatidylinositol 3,4,5-trisphosphate
PI(4)P	Phosphatidylinositol 4-phosphate

## Conflict of interest

Zhuo Ma, Md. Nur Islam, Tao Xu and Eli Song declare that they have no conflict of interest.
